# Enhanced joint pain in diabetic patients with knee osteoarthritis is associated with increased synovitis, synovial immune cell infiltration, and erythrocyte extravasation

**DOI:** 10.3389/fendo.2024.1477384

**Published:** 2024-10-14

**Authors:** Annett Eitner, Veronika Rutte, Ivan Marintschev, Gunther O. Hofmann, Hans-Georg Schaible

**Affiliations:** ^1^ Department of Trauma, Hand and Reconstructive Surgery, Experimental Trauma Surgery, Jena University Hospital, Friedrich Schiller University, Jena, Germany; ^2^ Institute of Physiology 1/Neurophysiology, Jena University Hospital, Friedrich Schiller University, Jena, Germany

**Keywords:** osteoarthritis, diabetes mellitus, synovial tissue, inflammation, erythrocyte extravasation, macrophages, mast cells, pain

## Abstract

**Objective:**

Diabetes mellitus (DM) is an important risk factor for the development of osteoarthritis (OA), increasing OA progression and OA pain. To gain insight into the underlying mechanisms of how DM exacerbates OA processes and OA pain, this study analyzed histological differences of synovial tissues from non-DM and DM patients with OA and correlated these differences with knee pain severity.

**Materials and methods:**

Synovial tissue was obtained from 12 non-DM and 10 DM patients with advanced knee OA who underwent total knee arthroplasty. Synovial inflammation was assessed using the Synovitis score developed by Krenn. The Knee Injury and Osteoarthritis Outcome Score (KOOS) was used to assess knee pain intensity and disability in OA patients. The number of mast cells, macrophages, nerve fibers, capillaries, larger vessels and erythrocyte extravasation were analyzed microscopically in histological and immunostained synovial sections from non-DM and DM patients. Association analyses were performed to determine associations between OA knee pain and synovial changes affected by DM.

**Results:**

Synovial tissue from OA patients with DM had a higher synovitis score, more erythrocyte extravasation, and contained higher numbers of mast cells and macrophages compared to non-DM patients. The number of capillaries and vessels in the lining/sublining layer of the synovial tissue was reduced in DM patients. OA patients with DM had more severe knee pain compared to non-DM patients. The KOOS pain score was associated with the synovitis score, the number of tissue macrophages, and the number of mast cells in the synovial tissue (adjusted for age, sex, and BMI). In addition, the erythrocyte extravasation score was associated with the KOOS pain score and with the synovitis score.

**Conclusion:**

The study suggests that increased OA progression and pain severity in patients with DM result from more pronounced synovitis and synovial vascular leakage and increased infiltration of macrophages and mast cells.

## 1 Introduction

Osteoarthritis (OA), the most common joint disease, is a complex disease with pathological changes in all articular tissues, including cartilage, synovial tissue, infrapatellar fat pad, ligaments, menisci, the joint capsule, and subchondral bone (1, 2). Inflammatory and destructive processes in various joint tissues result in synovial inflammation, cartilage and meniscal degeneration, subchondral bone sclerosis, osteophytes, bone marrow lesions, and tendon and ligament instability (1, 2). In particular, synovial inflammation is now considered to be a key pathophysiological process in OA (3) and is associated with OA progression, pain severity and symptoms (1, 3–5). Histological and MRI studies have shown that synovial inflammation is present in the majority of OA patients (3). In particular, the infrapatellar fat pad, which forms an anatomo-functional unit with the synovial membrane, is often inflamed and fibrotic in OA patients, is a source of inflammatory mediators, and is therefore actively involved in the pathophysiological processes of OA and pain (3, 6). Several OA risk factors such as obesity, diabetes mellitus (DM), meniscal and ligamentous injuries, and mechanical stress are also associated with synovial inflammation (1). In addition to the frequently reported association between synovitis and joint pain in OA patients (1, 5), this association has also been described in patients with traumatic meniscal injury (7). Importantly, several clinical studies have shown that DM increases the progression and incidence of OA (8), and OA patients with DM have higher levels of synovial inflammation and joint pain (9–11), but the underlying mechanisms are not clear.

Berenbaum et al. have developed a concept of DM-induced OA that includes proposed mechanisms that exacerbate OA processes (12), but detailed studies of how DM affects OA processes are rare. DM-induced low-grade systemic inflammation or hyperglycemia itself may affect the pathophysiological processes of OA by activating local cells in the synovial tissue thereby promoting inflammatory processes and cytokine release (12). Increased synovial inflammation has been reported in OA patients with DM (10, 11, 13). Mast cells and macrophages are present in the synovial tissue, and both cell types can be activated by various stimuli (14). Activated mast cells and macrophages can produce and release pro-inflammatory mediators such as interleukin-6 (IL-6), tumor necrosis factor (TNF), and IL-1β (15, 16), leading to activation of synoviocytes and chondrocytes. This can increase the production of matrix metalloproteinases (MMPs) and decrease the production of cartilage matrix substances, leading to an imbalance of catabolic and anabolic processes and ultimately to cartilage degeneration (12, 17). Increased numbers of mast cells and macrophages have been found in DM-affected organs, where they mediate diabetic complications (15, 16). Whether mast cells and macrophages play an important role in osteoarthritic joints of DM patients is unclear. Previous studies have shown that IL-6 is elevated in the synovial fluid of OA patients with DM (10), which can increase the release of MMP-1 and decrease the production of Pro-Collagen Type II in human chondrocytes (18). In addition, the concentration of IL-6 in synovial fluid correlates with the intensity of joint pain in OA patients (10, 19).

In several organs, DM induces endothelial dysfunction, increased capillary permeability, and microvascular changes that can lead to vascular leakage, macro/microvascular lesions, and destruction of the capillary network (20–22). This may result in inadequate oxygen and nutrient delivery, inducing hypoxic conditions, oxidative stress, and inflammation that can damage nerve fibers and lead to neuropathy in peripheral tissues (23). Whether DM also induces vascular damage in synovial tissue is unknown.

To gain more insight into the mechanisms of how DM affects synovial tissue in OA patients, this study analyzed DM-associated changes in synovial cell composition, nerve fiber network, capillary network, especially beneath the synovial lining layer, and the functional integrity of this capillary network. In addition, correlation analysis was used to determine whether the DM-related synovial changes found were associated with joint pain intensity.

## 2 Materials and methods

### 2.1 Patients

Synovial tissues were obtained from 22 patients (12 female/10 male) with end-stage knee OA undergoing total knee arthroplasty. Only patients with presumed idiopathic OA were included, patients with post-traumatic OA, bacteria-infected arthritis, rheumatoid disease and immunosuppressed patients were excluded. Patients were informed of the purpose of the tissue sampling and provided written informed consent after the nature of all examinations was fully explained. The study was approved by the Ethics Committee for Clinical Trials of the Friedrich-Schiller-University of Jena (Ethical Approval Number: 3966-12/13, Approval Date: January 23, 2014) and was conducted in accordance with the Declaration of Helsinki.

### 2.2 Clinical design

Demographic data (sex, age, and body mass index (BMI)) and medical history were documented for all patients prior to arthroplasty (patient parameters in **Table 1**). The mean age of the patients was 70 years (± 7.95 years, standard deviation). On the day before arthroplasty, blood was collected for standard routine laboratory measurements of C-reactive protein (CRP), and glycated hemoglobin (HbA1c). HbA1c determines the three-month average blood glucose level and was used as a diagnostic tool to identify undiagnosed DM (if HbA1c ≥6.5%) and pre-DM (if HbA1c ≥ 5.7% and <6.5%) (24). Patients were grouped into non-DM (if HbA1c < 5.7%) and DM patients based on known diagnosis of DM and the blood level of HbA1c (if HbA1c ≥6.5%). Patients with pre-DM were not included.

**Table 1:** Demographic and laboratory parameters of non-DM and DM patients.

	Non-DM n = 12	DM n = 10	P Value
Age (years)	71.3 ± 7.4	68.4 ± 8.6	0.582
Sex (female/male)	50%/50%	60%/40%	0.639^1^
BMI (kg/m²)	26.9 ± 3.0	32.8 ± 6.0	**0.009**
HbA1c (%)	5.4 ± 0.2	6.9 ± 0.6	**<0.001**
CRP (mg/l)	3.2 ± 1.9	6.1 ± 6.5	0.674

Data represent mean values ± SD or percentage recorded at the time of biopsy. P values were calculated using Mann-Whitney U test or Chi-Square test (^1^).

BMI, body mass index; CRP, C-reactive protein; HbA1c, glycated hemoglobin A1c. Bold entries mark P values < 0.05.

### 2.3 Quantification of joint pain

The Knee Injury and Osteoarthritis Outcome Score (KOOS) was used to quantify OA pain and symptoms in the week prior to surgery (25). The KOOS questionnaire included questions about OA symptoms such as swelling, stiffness, and restricted range of motion of the OA affected knee (KOOS symptoms), pain in the OA affected knee during certain activities (KOOS pain), and disability to perform activities of daily living (KOOS ADL) such as going shopping, getting in and out of the car, or doing homework. Each KOOS subscale score is calculated and converted to a 0-100 scale, with zero representing the worst outcome and 100 representing no knee problems.

### 2.4 Histological evaluation of synovial tissue

Synovial tissues from 4 different regions of the knee joint (suprapatellar recess, medial and lateral femoral regions, infrapatellar fat pad) were fixed with 4% paraformaldehyde (Sigma-Aldrich, Taufkirchen, Germany) in phosphate-buffered saline for 24 hours at 4°C, dehydrated, and embedded in Technovit 9100 methyl methacrylate (Heraeus Kulzer, Wehrheim, Germany) according to the manufacturer’s instructions. Synovial sections were cut from the polymerized Technovit 9100 blocks using a Polycut S microtome (Reichert-Jung, Heidelberg, Germany). Sections were treated with 2-methoxyethyl acetate (Merck KGaA, Darmstadt, Germany) to remove the polymer and rehydrated in decreasing concentrations of alcohol.

To evaluate the degree of inflammation, synovial sections were stained with Mayer’s hematoxylin and eosin G (Merck KGaA) and embedded in Histofluid (Marienfeld, Lauda-Königshofen, Germany). The inflammation of the synovial tissue was scored according to Krenn (26). This Synovitis score includes evaluation of the enlargement of the synovial lining layer, cellular density of the synovial stroma and pannus formation, and leukocyte infiltration as previously described (10, 26). The total synovitis score ranges from 0 to 9 (0-1: no synovitis; 2-3: low-grade, 4-6: medium-grade, 7-9: high-grade synovitis). Three selected areas of 1 x 0.5 mm per section were analyzed using an Axioplan2 microscope (Zeiss, Oberkochen, Germany) with a 20x dry and a 63x oil objective. The mean synovitis score per patient was calculated from the four different regions and the three selected areas per section.

### 2.5 Microscopic evaluation of mast cells, macrophages, innervation and vascularity

To visualize mast cells, synovial sections were stained with 1% Toluidine blue O (Merck KGaA) for 2 minutes and embedded in Histofluid (Marienfeld).

For immunohistochemical evaluation of macrophages, innervation, and vascularity, targets were labeled with anti-CD68 antibody (1:100, #ab955, abcam, Cambridge, UK), anti-PGP9.5 antibody (1:1000, AbD serotec, Düsseldorf, Germany), and anti-Collagen IV antibody (1:100, #MAS-14100, Thermo-Fisher Scientific, Waltham, MA, USA) after heat-induced antigen retrieval in citrate buffer (10 mM, pH 6.0) using an autoclave (120°C for 15 minutes). Isotype-specific Alexa 488-conjugated secondary antibodies (1:200, Invitrogen, Carlsbad, CA, USA) were used for immunofluorescence labeling of nerve fibers. Sections were counterstained with Hoechst 34580 (1:1000, Invitrogen) and embedded in ProLong Gold (Invitrogen). For immunohistochemical detection of macrophages and vessels, the Vectastain ABC detection system (Vector Laboratories, Burlingame, CA, USA) was used with biotinylated IgG antibodies in combination with an avidin/biotinylated enzyme complex and the enzyme substrate AEC. For a better visualization of target localization and morphology, the sections were counterstained with hemalaun (Merck KGaA) for 1 minute and embedded in glycerol gelatine (Merck KGaA). Negative controls did not exhibit nonspecific binding from the secondary antibodies or autofluorescence.

Mast cells, macrophages, innervation, and vascularity were analyzed and quantified in the three previously selected areas of 1 x 0.5 mm per section of the four different synovial regions using the Axioplan2 microscope with a 20x dry and a 63x oil objective equipped with an image analyzing system (Axiovision, Zeiss, Oberkochen, Germany). The number of mast cells and synovial tissue macrophages (excluding macrophage-like synoviocytes in the lining layer) was counted and the mean value per mm² was calculated for each category and each patient. Nerve fibers and vessels were analyzed separately according to their location in the lining/sublining layer (0-200 µm starting from the lining layer) and in deeper synovial tissue (>200 µm to < 500 µm starting from the lining layer) in the three selected areas. Vessels were additionally analyzed separately as capillaries and vessels according to their diameter as measured by the image analysis software (capillaries: d<10 µm, vessels: d≥10 µm).

In hemalaun (Merck KGaA)-stained synovial sections, the leakage of synovial vessels was assessed by determining the presence of extravasated erythrocytes near the vessels (yes = 1, no erythrocytes = 0). The erythrocyte extravasation score was calculated as the mean of the four different synovial samples and ranges from 0 to 1.

### 2.6 Statistical analysis

Statistical analyses were performed using SPSS statistics 27 software (SPSS, Inc, Chicago, IL, USA) and GraphPad Prism 10 software (Boston, MA, USA). Demographic and laboratory parameters of the included patients were expressed as mean ± standard deviation (SD) or percentage. Parameters of non-DM and DM patients were compared using the Mann-Whitney *U* Test or chi-squared test, respectively. Experimental data of non-DM and DM patients were presented as scatter dot plot. Differences between the two groups were analyzed using the Mann-Whitney *U* test. Effect sizes were expressed as Cohen’s *d* and calculated as the difference between the means divided by the pooled standard deviation. Correlation analyses were performed using the Spearman’s rank correlation coefficient *R*. Multivariate linear regression analysis was performed to analyze the association between the KOOS pain score and the histological parameters and adjusted for age, sex, and BMI. Significance was accepted at p < 0.05.

## 3 Results

### 3.1 Patients

This study included 12 non-DM and 10 DM patients with OA. **Table 1** shows the demographic data and laboratory blood parameters of both groups of patients. DM patients had significantly higher BMI and higher levels of HbA1c compared to non-DM patients. Age, sex and the level of CRP were comparable between both groups.

### 3.2 DM-related histological alterations of synovial tissue

**Figure 1:**
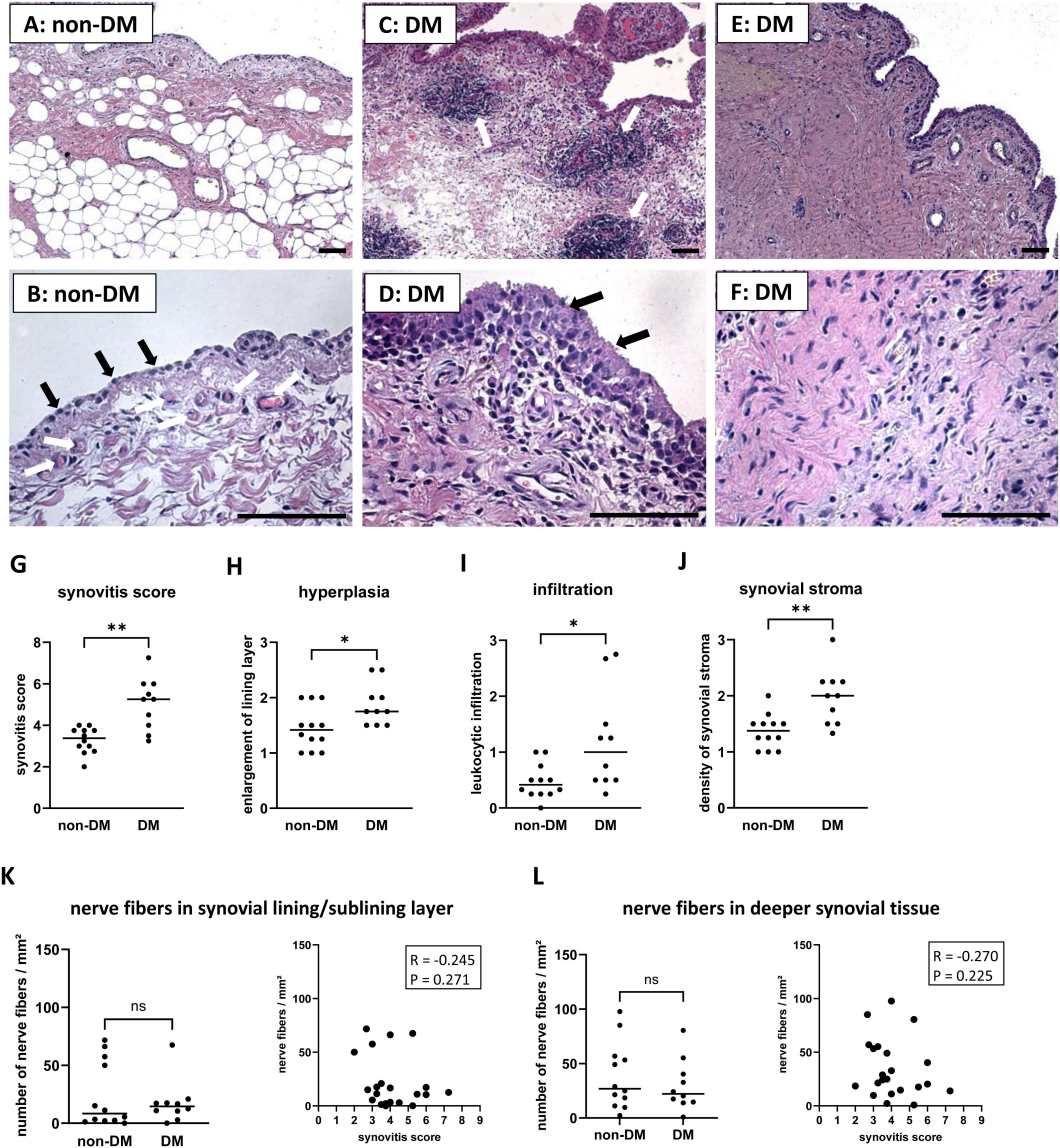
Histological differences in synovial tissue from non-DM and DM patients. Representative synovial sections from non-DM patients showing a normal appearance **(A)**, a continuous layer of lining cells (**B**, black arrows) and a network of capillaries (**B**, white arrows) embedded in loose connective tissue. Representative synovial sections from DM patients showing strong leukocytic infiltration (**C**, see arrows), marked hyperplasia (**D**, see arrows) and dense stroma **(E, F)**. Evaluation of the synovitis score **(G)** including the parameters hyperplasia **(H)**, leukocytic infiltration **(I)** and density of the synovial stroma **(J)** of non-DM (N = 12) and DM (N = 10) patients with osteoarthritis. Analysis of nerve fibers in the synovial lining/sublining layer **(K)** and in deeper synovial tissue **(L)** of non-DM (N = 12) and DM (N = 10) patients, and correlation analysis between the number of nerve fibers and the synovitis score. Statistical analysis between non-DM and DM patients: Mann-Whitney *U* test, *p < 0.05, **p< 0.01. Correlation analysis using Spearman rank correlation coefficient (R). Scale bars 100 µm.

Only few synovial sections from OA patients showed nearly normal synovial tissue without evidence of hyperplasia or leukocyte infiltration (**Figures 1A, B**). Normal, uninflamed synovial tissue consists of a continuous layer of synovial lining cells and, beneath this lining layer is a network of nerve fibers and capillaries embedded in loose connective tissue (4, 27, 28). Beneath this layer, the synovial tissue is composed of adipocytes, but often a band of fibrous tissue separates the synovial membrane from deeper adipose tissue (28). However, OA patients often have synovial tissue with clear signs of hyperplasia, leukocyte infiltration, and severe synovial fibrosis. These signs of synovial inflammation were present in the synovial tissue of both non-DM and DM patients, but were more pronounced in DM patients (examples of these parameters are shown in **Figures 1C–F**). OA patients with DM had a significantly higher synovitis score (Effect size *d* = 1.85, p = 0.002), including more hyperplasia, more leukocyte infiltration, and higher density of synovial stroma compared to non-DM patients (**Figures 1G–J**). However, analysis of nerve fibers in the lining/sublining layer and deeper layers of synovial tissue showed no significant difference between the two patient groups, and the number of nerve fibers was not associated with the synovitis score (**Figures 1K, L**).

**Figure 2:**
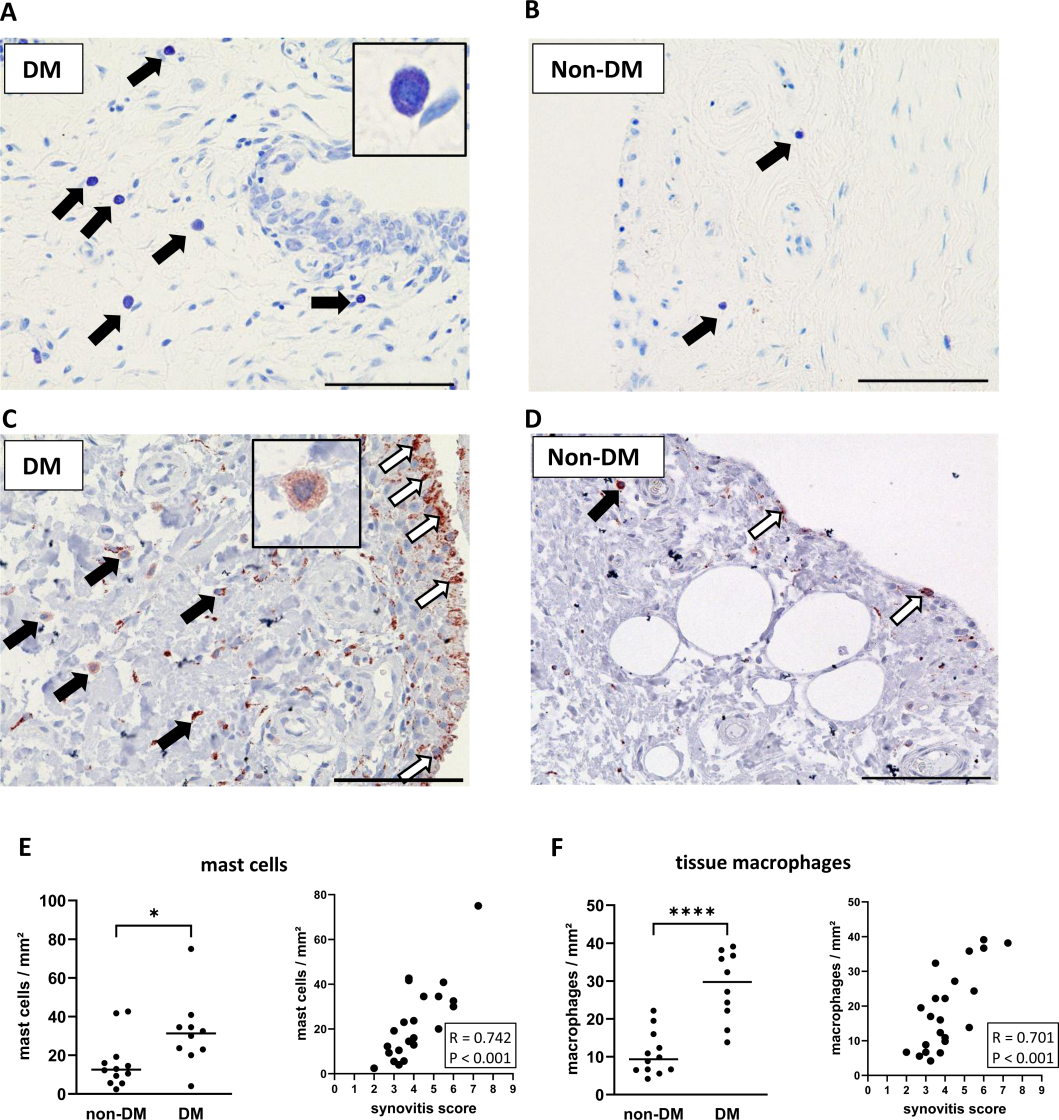
Histological differences in synovial tissue from non-DM and DM patients. Representative synovial sections **(A, B)** from DM and non-DM patients with osteoarthritis showing Toluidine blue-labeled mast cells. Mast cell granules appear dark purple: black arrows. Representative synovial sections **(C, D)** showing immuno-stained macrophages (brown color; tissue macrophages: black arrows, synovial-like macrophages: white arrows). Analysis of the number of mast cells **(E)** and tissue macrophages **(F)** in non-DM (N = 12) and DM (N = 10) patients. Correlation analysis between synovitis score and number of mast cells **(E)** and number of tissue macrophages **(F)**. Statistical analysis between non-DM and DM patients: Mann-Whitney *U* test, *p < 0.05, ****p < 0.0001. Correlation analysis by Spearman rank correlation coefficient (R). Scale bars 100 µm.

Stained mast cells and macrophages in synovial sections from DM and non-DM patients with OA are shown in **Figure 2**. Synovial tissue from DM patients showed higher numbers of mast cells (*d* = 1.01, p = 0.030, **Figures 2A, E**) and tissue macrophages (*d* = 2.40, p < 0.001, **Figures 2C, F**), and both mast cell and tissue macrophage numbers were associated with synovitis score (**Figure 2E**: mast cells vs. synovitis score, R = 0.742 confidence interval (CI): 0.459, 0.884, p < 0.001; **Figure 2F**: tissue macrophages vs. synovitis score, R = 0.701 CI: 0.340, 0.889, p < 0.001).

**Figure 3:**
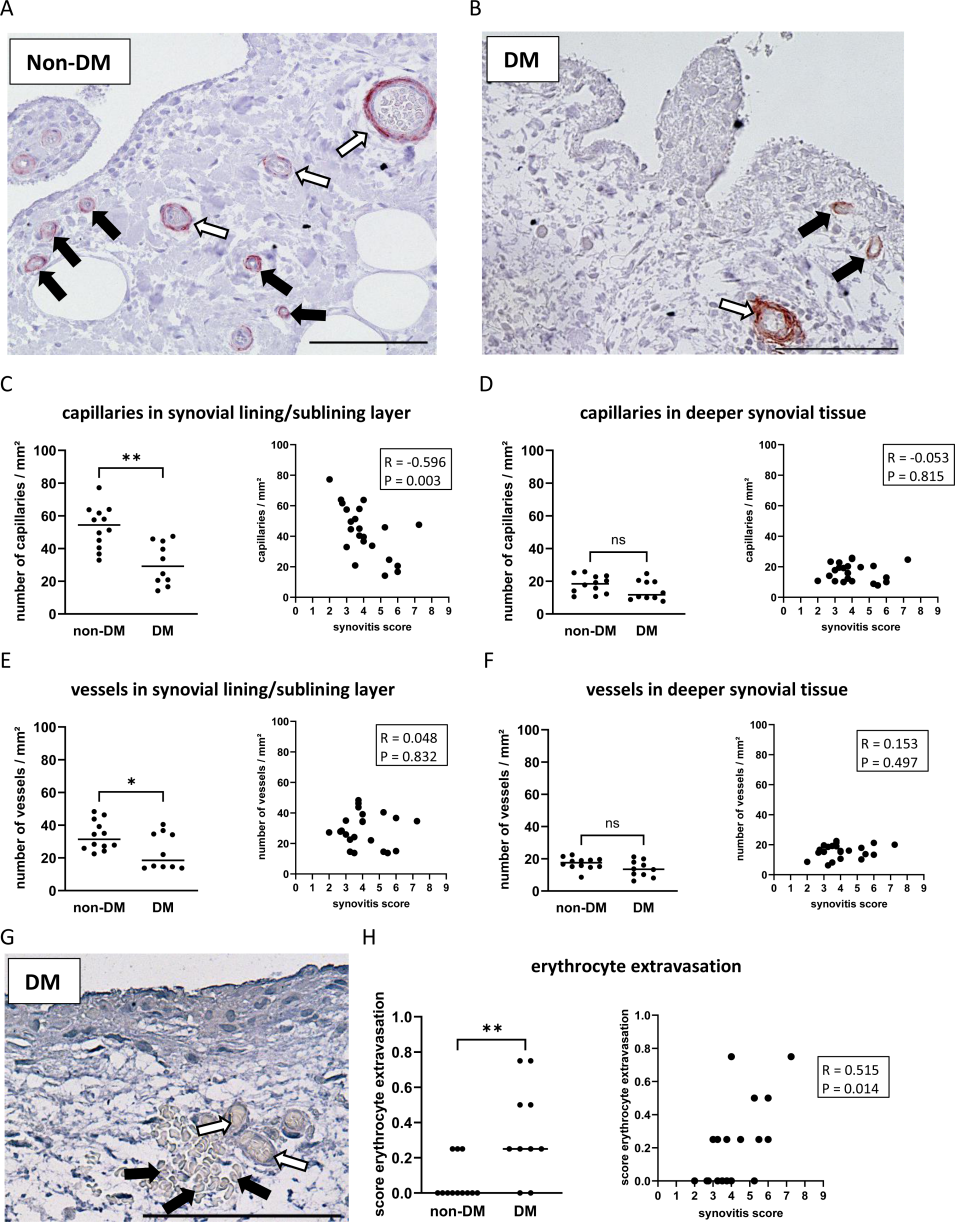
Histological differences in synovial tissue from non-DM and DM patients. Representative synovial sections **(A, B)** showing immuno-stained capillaries (black arrows) and vessels (white arrows) in the synovial lining/sublining layer. Analysis of capillaries in the synovial lining/sublining layer **(C)** and in deeper synovial tissue **(D)**, vessels in the synovial lining/sublining layer **(E)** and in deeper synovial tissue **(F)**, and analysis of erythrocyte extravasation **(G, H)** in non-DM (N = 12) and DM (N = 10) patients with osteoarthritis. **(G)** histological image of synovial tissue showing erythrocytes (black arrows) outside of blood vessels (white arrows) as a feature of synovial vascular leakage. Statistical analysis between non-DM and DM patients: Mann-Whitney *U* test, ns: not significant, *p < 0.05, **p< 0.01. Correlation analysis by Spearman rank correlation coefficient (R). Scale bar 100 µm.

A high number of capillaries and larger vessels was often found in the lining/sublining layer in non-DM patients (**Figure 3A**), but the number was significantly decreased in DM patients (**Figures 3B, C, E** *d* = 1.73, p = 0.002). Higher synovial inflammation was associated with a lower number of capillaries in the lining/sublining layer (R = -0.596 CI: -0.878, -0.158, p = 0.003), whereas capillaries or larger vessels in deeper layers were neither reduced in OA patients with DM nor associated with synovitis score (**Figures 3C–F**). Assessment of synovial vascular leakage by the presence of erythrocytes in the synovial tissue outside the blood vessels (**Figure 3G**) showed that 50% of all patients had erythrocyte extravasation in the synovial tissue. In non-DM patients, only 25% of the synovial tissue showed erythrocyte extravasation, whereas in the DM group, erythrocyte extravasation was found in the synovial tissue of 80% of the patients (Chi-squared test non-DM vs. DM: p = 0.01). The calculated erythrocyte extravasation score per patient (mean of all synovial tissues analyzed) showed that DM patients had a significantly higher erythrocyte extravasation score compared to non-DM patients (*d* = 2.57, p = 0.005, **Figure 3H**). The erythrocyte extravasation score correlated with increased synovial inflammation (**Figure 3H**, R = 0.515 CI: 0.113, 0.785, p = 0.014).

To evaluate whether histological synovial parameters were affected by patient parameters such as sex, age, and BMI, or by blood levels of HbA1c and CRP, a correlation analysis was performed and is presented in **Table 2**. None of the synovial parameters analyzed were significantly affected by sex, age, and CRP levels. A significant correlation was found only between BMI and tissue macrophages, all other synovial parameters were not affected by the BMI of the patients. The HbA1c level was positively correlated with the grade of synovitis, the number of tissue macrophages, and the erythrocyte extravasation score (**Table 2**). A negative correlation was found between the HbA1c level and the number of capillaries in the lining/sublining layer.

**Table 2:** Correlation analysis of synovial parameters with sex, age, BMI, HbA1c and CRP.

		Sex	Age	BMI	HbA1c	CRP
Synovitis	R p	0.253 0.256	-0.084 0.709	0.353 0.108	**0.705** **<0.001**	0.315 0.153
Mast cells	R p	0.094 0.679	-0.363 0.096	0.144 0.523	0.340 0.131	0.158 0.482
Tissue macrophages	R p	0.396 0.068	-0.013 0.955	**0.436** **0.043**	**0.714** **<0.001**	0.419 0.052
Capillaries lining/sublining layer	R p	-0.144 0.523	-0.134 0.552	-0.343 0.118	**-0.702** **<0.001**	-0.034 0.879
Erythrocyte extravasation	R p	-0.047 0.835	-0.155 0.492	0.143 0.525	**0.481** **0.027**	0.130 0.566

Data represent correlation coefficient R and p values calculated using Spearman’s rank correlation.

BMI, body mass index; HbA1c, glycated hemoglobin A1c; CRP, C-reactive protein. Bold entries mark P values < 0.05.

### 3.3 Correlation of DM-related synovial alterations with joint pain intensity

**Figure 4:**
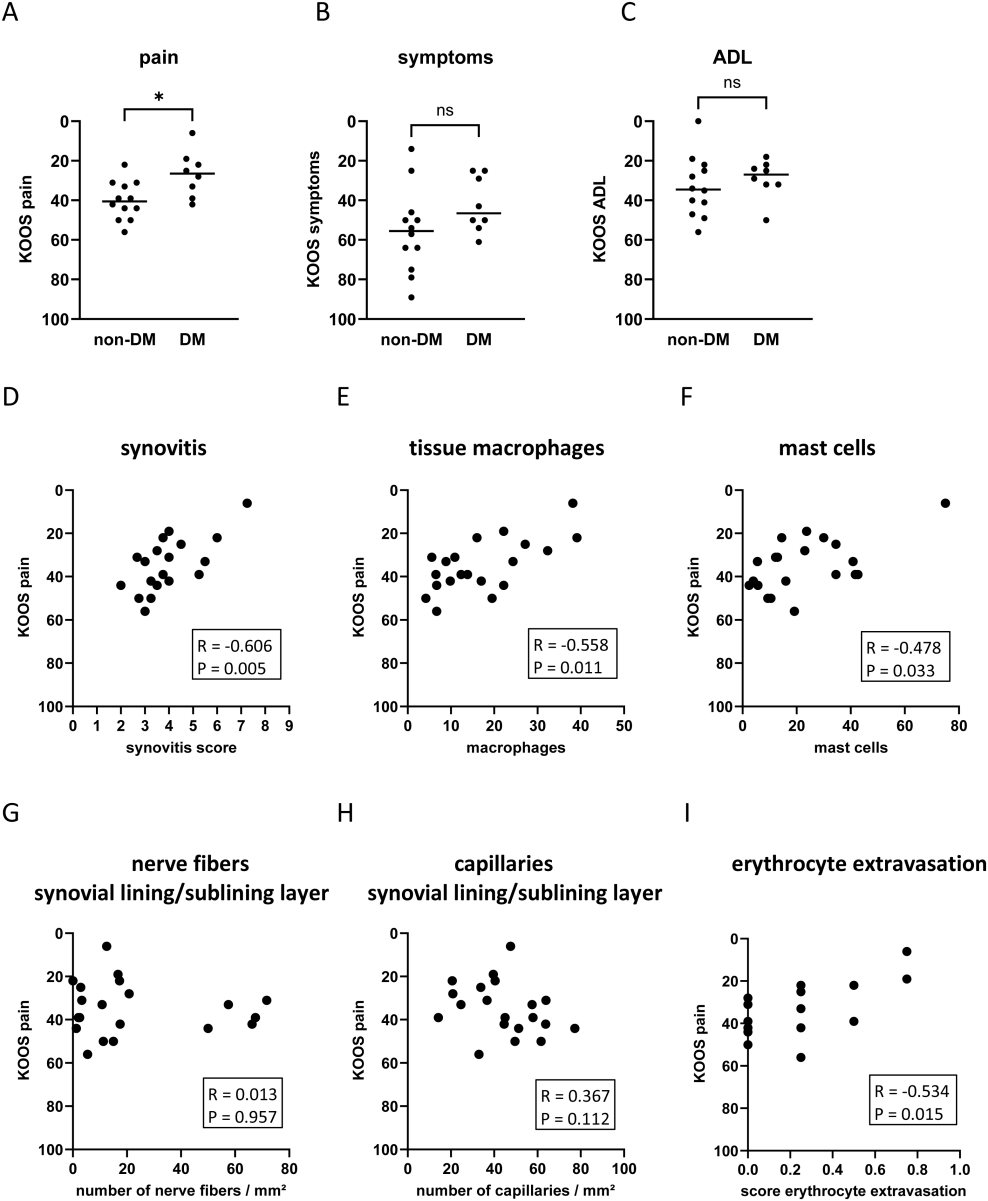
Impact of DM on KOOS pain **(A)**, KOOS symptoms **(B)**, and KOOS ADL **(C)** in non-DM (N = 12) and DM (N = 10) patients with osteoarthritis. Correlation analysis between KOOS pain and synovitis score **(D)**, tissue macrophages **(E)**, mast cells **(F)**, number of nerve fibers in the synovial lining/sublining layer **(G)**, capillaries in the synovial lining/sublining layer **(H)**, and score erythrocyte extravasation **(I)**. KOOS score = 0 represents the worst outcome and KOOS score = 100 represents no knee problems. Statistical analysis between non-DM and DM patients: Mann-Whitney *U* test, ns, not significant, *p < 0.05. Correlation analysis by Spearman rank correlation coefficient (R).

OA patients with DM had significantly more knee pain compared to non-DM patients (**Figure 4A**, *d* = 1.28, p = 0.018). Symptoms and disability were not significantly different between the two patient groups (**Figures 4B, C**). Higher knee pain intensity was associated with increased synovial inflammation (**Figure 4D**: Synovitis score vs. KOOS pain: R = -0.606 CI: -0.843, -0.207, p = 0.005) and increased numbers of tissue macrophages and mast cells (**Figure 4E**: tissue macrophages vs. KOOS pain: R = -0.558 CI: -0.823, -0.131, p = 0.011; **Figure 4F**: mast cells vs. KOOS pain: R = -0.478 CI: -0.745, -0.096, p = 0.033). The KOOS pain score was not significantly associated with the number of nerve fibers or capillaries in the lining/sublining layer (**Figures 4G, H**). However, the pain intensity correlated significantly with erythrocyte extravasation (**Figure 4I**: R = -0.534 CI: -0.816, -0.054, p = 0.015).

Multivariate regression analysis was performed to assess whether these associations were influenced by patient parameters (**Table 3**). The associations between the KOOS pain score and histological parameters were similar in the unadjusted model 1 and after adjustment for age, sex, and BMI (**Table 3**). In all models analyzed, the KOOS pain score was significantly associated with the synovitis score, the number of tissue macrophages, the number of mast cells, and erythrocyte extravasation score. Hyperplasia of the synovial lining layer was the most important parameter within the synovitis score in terms of association with pain intensity. When comparing the standardized Beta of all analyzed parameters in model 3, the number of tissue macrophages showed the strongest association with the KOOS pain score (**Table 3**, standardized Beta = -0.781).

**Table 3:** Association between the KOOS pain score and histological parameters using multivariate linear regression analysis.

Model adjusted for	Model 1 unadjusted		Model 2 age and sex		Model 3 age, sex and BMI		stand. Beta
	B (95% Cl)	P	B (95% Cl)	P	B (95% Cl)	P	
Synovitis-Score	-6.41 (-10.05, -2.77)	**0.002**	-6.45 (-10.37, -2.52)	**0.003**	-6.86 (-10.93, -2.80)	**0.003**	**-0.704**
Hyperplasia of lining layer	-21.23 (-31.77, -10.70)	**<0.001**	-21.49 (-32.23, -10.75)	**0.001**	-21.83 (-32.92, -10.75)	**0.001**	**-0.726**
Leukocyte infiltration	-8.15 (-15.03, -1.27)	**0.023**	-8.58 (-16.05, -1.12)	**0.027**	-9.52 (-17.42, -1.61)	**0.022**	**-0.591**
Synovial stroma	-12.24 (-26.50, 2.03)	0.088	-11.56 (-27.93, 4.81)	0.154	-11.66 (-28.67, 5.35)	0.165	-0.373
Tissue macrophages	-0.75 (-1.17, -0.33)	**0.001**	-0.80 (-1.25, -0.35)	**0.002**	-0.89 (-1.34, -0.43)	**0.001**	**-0.781**
Mast cells	-0.38 (-0.66, -0.10)	**0.010**	-0.39 (-0.72, -0.06)	**0.022**	-0.39 (-0.73, -0.05)	**0.027**	**-0.577**
Erythrocyte extravasation	-30.83 (-49.37, -12.30)	**0.003**	-6.45 (-10.37, -2.52)	**0.003**	-31.80 (-52.78, -10.82)	**0.006**	**-0.656**

KOOS, Knee injury and Osteoarthritis Outcome Score; BMI, body mass index; 95% Cl, 95% confidence interval; B, regression coefficient B; stand. Beta, standardized coefficient Beta. Bold entries mark P values < 0.05.

## 4 Discussion

Diabetes affects OA progression and OA knee pain. Our data show DM-related changes in the synovial cellular composition, the capillary network, especially beneath the synovial lining layer, and the integrity of this capillary network, as well as the association of these changes with OA pain intensity. Synovial tissue from OA patients with DM showed a higher inflammation and impaired capillary network in the lining/sublining layer. In addition, OA patients with DM had a higher knee pain severity compared to non-DM patients. The KOOS pain score was associated with the DM-affected parameters synovitis score, number of tissue macrophages, number of mast cells, and score of erythrocyte extravasation (adjusted for age, sex, and BMI).

Our data confirm the results of previous work that OA patients with DM have a higher synovitis score and OA pain compared to non-DM patients (10, 11, 13). High levels of glucose and advanced glycation end products (AGE) in the serum of DM patients can directly induce the activation of inflammatory pathways and the production of reactive oxygen species (ROS) and vascular endothelial growth factor (VEGF) in synovial fibroblasts (29–31). AGE-induced production and release of IL-6, prostaglandin E_2_, and TNF can further induce inflammatory processes in other cell types and increase neuronal activity to mechanical stimuli (30–32). Since DM increases the concentration of AGEs in the synovial fluid of OA patients (33), DM-induced AGEs may also induce the release of inflammatory mediators in other joint compartments such as cartilage (34). The use of an AGE inhibitor has been shown to reduce serum AGE concentrations and significantly improve pain scores and inflammation in OA patients (35).

In addition to increased synovitis, our results show higher numbers of mast cells and tissue macrophages in the synovial tissue of DM patients. Both cell types are increased in the synovial tissue of OA patients (14), but our analysis shows that the comorbidity DM further increases the infiltration of the synovial tissue with these cells, which is consistent with reported DM complications in other organs (15, 16). Infiltration with tissue macrophages is a common feature of DM complications, such as neuropathy, retinopathy, atherosclerosis and nephropathy (15), and the release of substances by macrophages contributes to inflammation and macrophage-mediated complications (15). In peripheral neuropathy, the prominent presence of macrophages in perivascular lesions is associated with demyelination of nerve fibers (15). In retinopathy, macrophages are involved in the pathological processes of microvascular cell apoptosis, neovascularization, and fibrosis (15). Diabetic conditions, such as high serum glucose, AGEs, and oxidized low-density lipoprotein stimulate macrophage migration, activation, and production of cytokines, ROS and MMPs (15). These released substances are also involved in the induction and progression of OA (17) as well as in pain mechanisms (5, 10, 36). Our results clearly show an association of macrophages with synovial inflammation and OA pain intensity. Thus, DM-mediated macrophage accumulation in synovial tissue may be a cause of increased OA progression and OA pain in patients with DM.

Mast cells are mostly present just beneath the synovial lining layer and often near blood vessels and nerve fibers (14, 37, 38). The number of mast cells is increased in the synovial tissue of OA patients and sometimes even higher than in RA patients (14, 39). By releasing cytokines such as IL-6 and TNF, mast cells contribute to inflammatory and many degenerative processes, while interacting with fibroblasts, synoviocytes, blood vessels and nerve fibers (16, 38). Our data show a strong association between the prevalence of mast cells and the synovitis score. Although the number of mast cells can be comparable between early and late OA, it correlates with radiographic OA changes (38, 39). Mast cells can release numerous growth factors such as VEGF and nerve growth factor (NGF), which promote angiogenic processes and affect nerve fibers (38). Even in normal tissues, mast cells show a strong interaction with nerve fibers, as neurotransmitters can activate them and substances released by mast cells can influence neuronal activity (37, 38). The analysis of our data shows an association between the number of mast cells in the synovial tissue and the KOOS pain score, even after including age, sex and BMI as confounders. Although mediators released by mast cells are known to sensitize neurons, cause OA joint pain, and promote neuropathic pain (5, 32, 36, 38, 40, 41), de Lange-Brokaar et al. could not confirm an association between the number of mast cells and self-reported OA pain (39). Increased mast cell infiltration has also been reported in DM complications such as nephropathy, but the underlying mechanism by which mast cells mediate diabetic nephropathy is unknown (16). The higher number of mast cells in the synovial tissue of DM patients may contribute to the increased inflammatory processes and pain in these patients.

Beside inflammation, chronic hyperglycemia affects the microvasculature of several organs and peripheral tissues (20). DM-induced microvascular complications such as nephropathy, retinopathy, and neuropathy have been extensively studied and described (20–22), but DM-induced microvascular alterations in the synovial tissue have not been investigated. Our data show for the first time the impaired capillary network in the synovial tissue of DM patients. The high number of capillaries, which can be normally found in the lining/sublining layer of healthy synovial tissue (27, 28), was strongly reduced in patients with DM. Capillaries in the deeper region of the synovial tissue were not significantly affected by DM. In addition to the reduced capillary network, higher erythrocyte extravasation in the synovial tissue was found in DM patients. Since both parameters correlated with the HbA1c levels, hyperglycemia may be the reason for these changes. Hyperglycemia activates several metabolic pathways, which trigger cellular oxidative stress, inflammation, and mitochondrial dysfunction (20). As a result, hyperglycemia affects endothelial cells and pericytes, induces apoptosis, and can lead to loss of endothelial cells and pericytes, and increased capillary permeability (20). Increased capillary permeability has long been recognized as an early phenomenon in diabetes (21). Hyperglycemia itself or the DM-induced increased systemic inflammation can affect the endothelial barrier function (42, 43). Important cytokines elevated in DM-induced systemic inflammation, such as IL-6 (44, 45), lead to a loss of endothelial intercellular adhesion and cause a breakdown of the vascular barrier (42). IL-6 at concentrations found in the serum of DM individuals also leads to a reduced capillary network formation (44). Particularly in diabetic retinopathy, the impairment and loss of pericytes results in immature vessels, which are fragile, permeable, and can easily rupture, leading to erythrocyte extravasation (46, 47). In addition to the well-documented degeneration of capillaries in the retina and periphery, DM also induces reduced capillary density and pericyte loss in the myocardium (48). Erythrocyte extravasation can be found in various types of vascular lesions, especially in atherosclerotic lesions, and is associated with the progression of vascular disease (49, 50). Under diabetic conditions, glycated erythrocytes impair endothelial function and promote rupture of atherosclerotic plaques (50). Vessels in synovial tissue may also respond to inflammatory conditions with a loss of pericytes, thus a significant occurrence of immature vessels without pericyte recruitment has been found in synovial tissue of patients with inflammatory arthritis and rheumatoid arthritis (51, 52). Their occurrence in the synovial tissue increased with higher disease activity and severity, and greater inflammatory cell infiltration, and decreased in response to anti-TNF therapy (51). Synovial tissue from 24% of patients with rheumatoid arthritis shows erythrocyte extravasation (53). In addition, immature vessels were found in the synovial tissue of 21% of OA patients, whereas no immature vessels were found in normal synovial tissue (51). Synovial vascular leakage and erythrocyte extravasation may be an important microvascular complication of DM in OA patients, that may activate pro-inflammatory processes in synovial cells and may increase OA progression.

In addition to these complications, nerve fibers in DM patients may also be affected by these pathological processes. A reduction of nerve fibers in relation to the synovial inflammation has been described in the synovial tissue of OA patients (27), but in our present study, significant differences in the number of nerve fibers in the different regions of the synovial tissue were not found between non-DM and DM patients.

Interestingly, neither the number of capillaries nor the number of nerve fibers in the lining/sublining layer of the synovial tissue correlated with OA pain, whereas the score of erythrocyte extravasation significantly correlated with the KOOS pain score in OA patients. As a result of microvascular leakage or rupture, inflammatory mediators such as IL-6 or AGE can easily enter the synovial tissue from the plasma and directly affect surrounded nerve fibers or stimulate synovial fibroblast, macrophages or mast cells to release additional pain mediators. IL-6 and AGE can bind to receptors on nerve fibers, increase neuronal activity leading to increased pain sensation (32, 54).

Limitations of the present study are that the results are limited to patients with end-stage OA and that the causality of the associations found cannot be tested in this study. Previous studies have shown that DM patients with a Kellgren/Lawrence grade I and II also have higher joint pain compared to non-DM patients (9), suggesting similar mechanisms in early- and end-stage OA. However, the present study shows clear morphological differences in synovial tissue between DM and non-DM patients and thus may trigger further research to determine whether targeting specific cells may reduce the risk of increased OA progression and pain severity in DM patients.

## 5 Conclusion

OA patients with DM show persistent and exaggerated alterations in the synovial tissue that are known to accelerate OA processes and OA pain. Hyperglycemia, DM-induced systemic inflammation or AGEs may directly induce microvascular alterations, migration of macrophages, and inflammatory processes in the synovial tissue of OA patients. All these parameters are strongly associated with pain intensity, but the number of tissue macrophages showed the strongest effect. Mast cells can further accelerate synovitis and may also be involved in the increased OA pain in DM patients. The inhibition of tissue macrophages may be an potential treatment to reduce OA pain and OA progression in patients with DM. The results of the study suggest that increased OA progression and pain in patients with DM result from more pronounced synovitis, enhanced macrophage and mast cell infiltration and increased synovial vascular leakage.

## Data Availability

The original contributions presented in the study are included in the article/supplementary material. Further inquiries can be directed to the corresponding author.
